# The Acinar Cage: Basement Membranes Determine Molecule Exchange and Mechanical Stability of Human Breast Cell Acini

**DOI:** 10.1371/journal.pone.0145174

**Published:** 2015-12-16

**Authors:** Aljona Gaiko-Shcherbak, Gloria Fabris, Georg Dreissen, Rudolf Merkel, Bernd Hoffmann, Erik Noetzel

**Affiliations:** Institute of Complex Systems ICS7: Biomechanics, Forschungszentrum Jülich, Jülich, Germany; Florida International University, UNITED STATES

## Abstract

The biophysical properties of the basement membrane that surrounds human breast glands are poorly understood, but are thought to be decisive for normal organ function and malignancy. Here, we characterize the breast gland basement membrane with a focus on molecule permeation and mechanical stability, both crucial for organ function. We used well-established and nature-mimicking MCF10A acini as 3D cell model for human breast glands, with ether low- or highly-developed basement membrane scaffolds. Semi-quantitative dextran tracer (3 to 40 kDa) experiments allowed us to investigate the basement membrane scaffold as a molecule diffusion barrier in human breast acini *in vitro*. We demonstrated that molecule permeation correlated positively with macromolecule size and intriguingly also with basement membrane development state, revealing a pore size of at least 9 nm. Notably, an intact collagen IV mesh proved to be essential for this permeation function. Furthermore, we performed ultra-sensitive atomic force microscopy to quantify the response of native breast acini and of decellularized basement membrane shells against mechanical indentation. We found a clear correlation between increasing acinar force resistance and basement membrane formation stage. Most important native acini with highly-developed basement membranes as well as cell-free basement membrane shells could both withstand physiologically relevant loads (≤ 20 nN) without loss of structural integrity. In contrast, low-developed basement membranes were significantly softer and more fragile. In conclusion, our study emphasizes the key role of the basement membrane as conductor of acinar molecule influx and mechanical stability of human breast glands, which are fundamental for normal organ function.

## Introduction

The pre-pubertal and pubertal mammary gland consists of ducts terminated by highly proliferative structures (called terminal end buds) without hollow lumen [1]. Milk secreting, lumen harboring alveoli are formed only during pregnancy as final functional developmental stage. The glandular unit is composed of 15–20 lobes of inner polarized luminal cells, an outer layer of contractile myoepithelial cells and is surrounded by a continuous basement membrane (BM)-scaffold [2–4]. BMs are highly organized and condensed extracellular matrix structures secreted by epithelial, endothelial and smooth muscle cells [5,6]. BM consist of a collagen IV mesh anchored to laminins, nidogen, entactin and perlecan [7]. The overall composition of BMs varies according to developmental stages and organ function [8]. BMs form physical barriers separating epithelial and stromal cells [9] and regulate molecule diffusion [2,3].

To date, trans-BM diffusion properties of human breast gland tissue remain unclear, but could play a fundamental role in excluding mid to large macromolecules, as it has been shown in-depth for the glomerular BM network [7,10]. The breast gland is a mechanically active tissue, where the BM plays a pivotal role for force homeostasis and mechanical stress shielding [11]. This balance is postulated to be deregulated in breast cancer development and to contribute to aggressive phenotypes. Expansive loads of a hyperproliferating tumor mass could cause a progressive BM thinning and breakdown [12]. In fact, the BM is a major mechanical barrier through which a tumor cell has to penetrate in order to invade the adjacent tissue [3,13]. Biophysical key processes such as altered substrate permeation and structural stability loss could hence fundamentally influence breast cancer development and invasion.

In the last decades considerable progress has been made regarding the understanding of tumor cell invasion [14]. Nevertheless, some particularly relevant biophysical aspects of this process are still insufficiently understood. Here, three-dimensional (3D) breast cancer *in vitro* models have emerged as a powerful tool for a wide range of cancer research applications [15]. The non-transformed human mammary epithelial cell line MCF10A, when grown on Engelbreth Holm Swarm (EHS) hydrogel, forms multicellular acini that feature key aspects of *in vivo* breast gland morphology, including BM deposition and lumen formation [16,17]. Although impressive studies have characterized the morphogenesis of the MCF10A breast gland model [17,18], there is still a lack of information regarding the actual role of the BM shell for mechanical homeostasis and organ function. Elucidation of these mechanisms is necessary for a better understanding of the physiology of healthy and tumorous breast gland tissue and thus for potentially contributing to more efficient therapeutic approaches.

Therefore we aimed to quantitatively characterize the human breast gland BM with respect to two fundamental functions: substrate permeation and mechanical support. In order to meet our requirements for a reliable biophysical BM characterization we aimed to minimize the morphological heterogeneity of MCF10A acini. The influence of epidermal growth factor (EGF) on acinar growth and differentiation was analyzed, in order to achieve high numbers of highly-differentiated acini with homogeneously developing BM scaffolds. Based on this, we characterized the BM as a permeation regulator by performing size-dependent dextran tracer experiments on acini with increasing BM development and focused on the role of collagen IV as structure-lending protein. In addition, we quantified the compressive force resistance properties of differentially developed BM scaffolds by atomic force microscopy (AFM) to improve the understanding of the mechanical function of this crucial cell-matrix associated meshwork for breast gland stability.

## Results

### Acinar differentiation can be triggered by fine-tuned EGF stimulation

**Fig 1:**
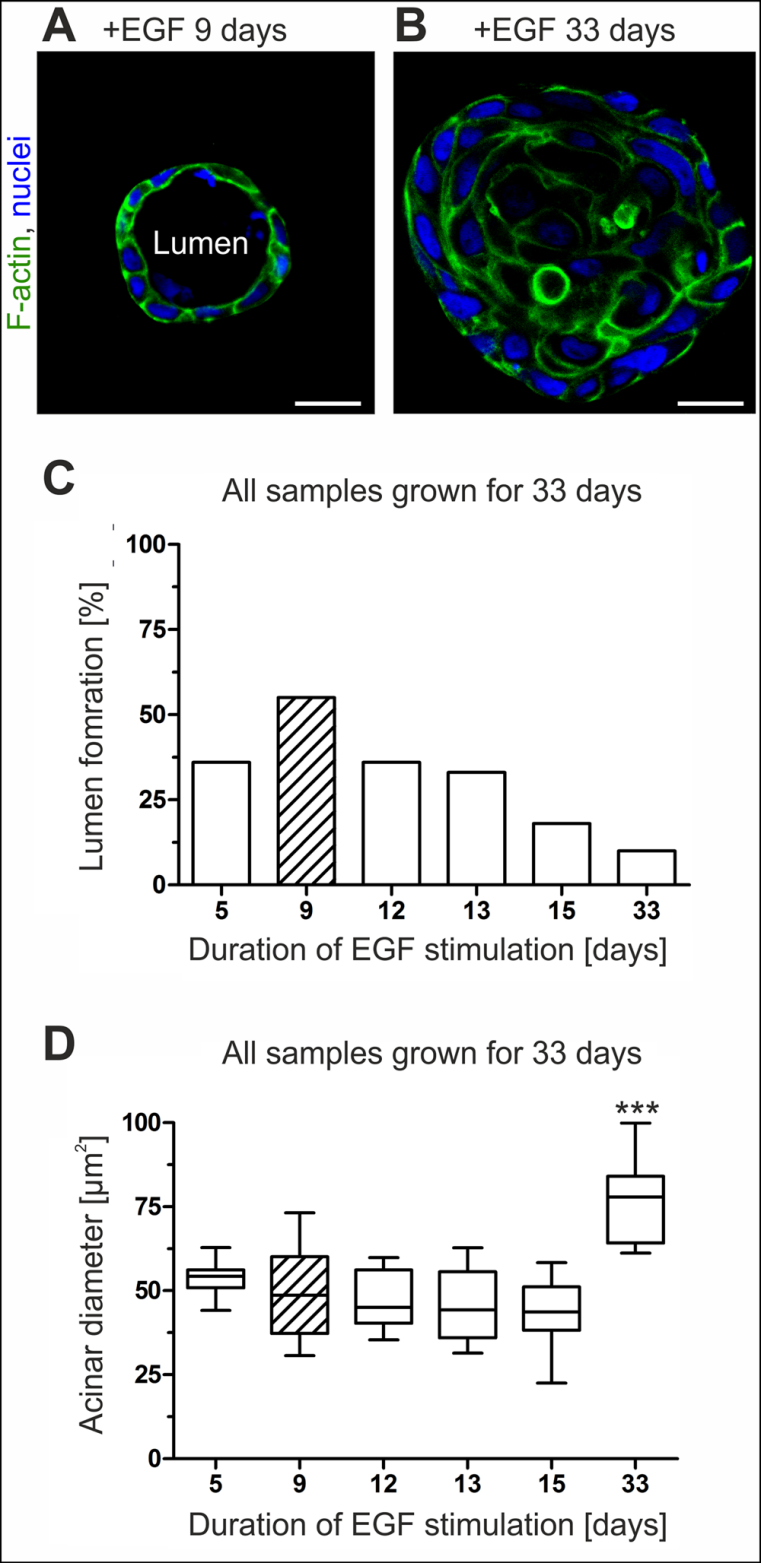
MCF10A lumen clearance and acini size are EGF triggered. Representative confocal images through the equatorial plane of acini illustrate the influence of EGF on acinar morphology. **A.** Acini grown for 33 days were stimulated with EGF for the first 9 days. **B.** A comparative sample with continuous EGF supplementation for 33 days. Scale bars = 20 μm. **C.** Plot illustrates the relation between lumen formation frequency and temporal EGF stimulation. **D.** Box plot analysis shows the relation of acini diameter to temporal EGF supplementation. At least 10 spheres were analyzed per sample. Horizontal lines: group medians. Boxes: 25–75% quartiles. Vertical lines: range, peak and minimum. Hatched bars indicate best combinatory effect size. ***: p < 0.0001.

As prerequisite for our biophysical characterization of the breast gland BM we intended to reduce the inherent morphological heterogeneity of developing MCF10A 3D cultures. We aimed to achieve higher fraction of MCF10A acini with homogeneously shaped and highly-developed BM shells. To this end we focused on EGF as main trigger for epithelial cell growth (Fig 1A and 1B). The influence of temporal staggered EGF withdrawal on morphogenesis was systematically tested on developing MCF10A acini (Fig 1C). Continuous EGF stimulation (5 ng mL^-1^) over a cultivation period of 33 days resulted in poor lumen formation frequency (10%). In contrast, early EGF withdrawal after 9 days resulted with 55% in the best lumen formation frequency (Fig 1C). Additionally, steady EGF supplementation over 33 days resulted in continuous cell proliferation and caused significantly increased mean acini diameters (77 μm, s.d. = 12 μm) compared to acini that experienced EGF depletion on day 9 (49 μm, s.d. = 13 μm) (Fig 1D).

**Fig 2:**
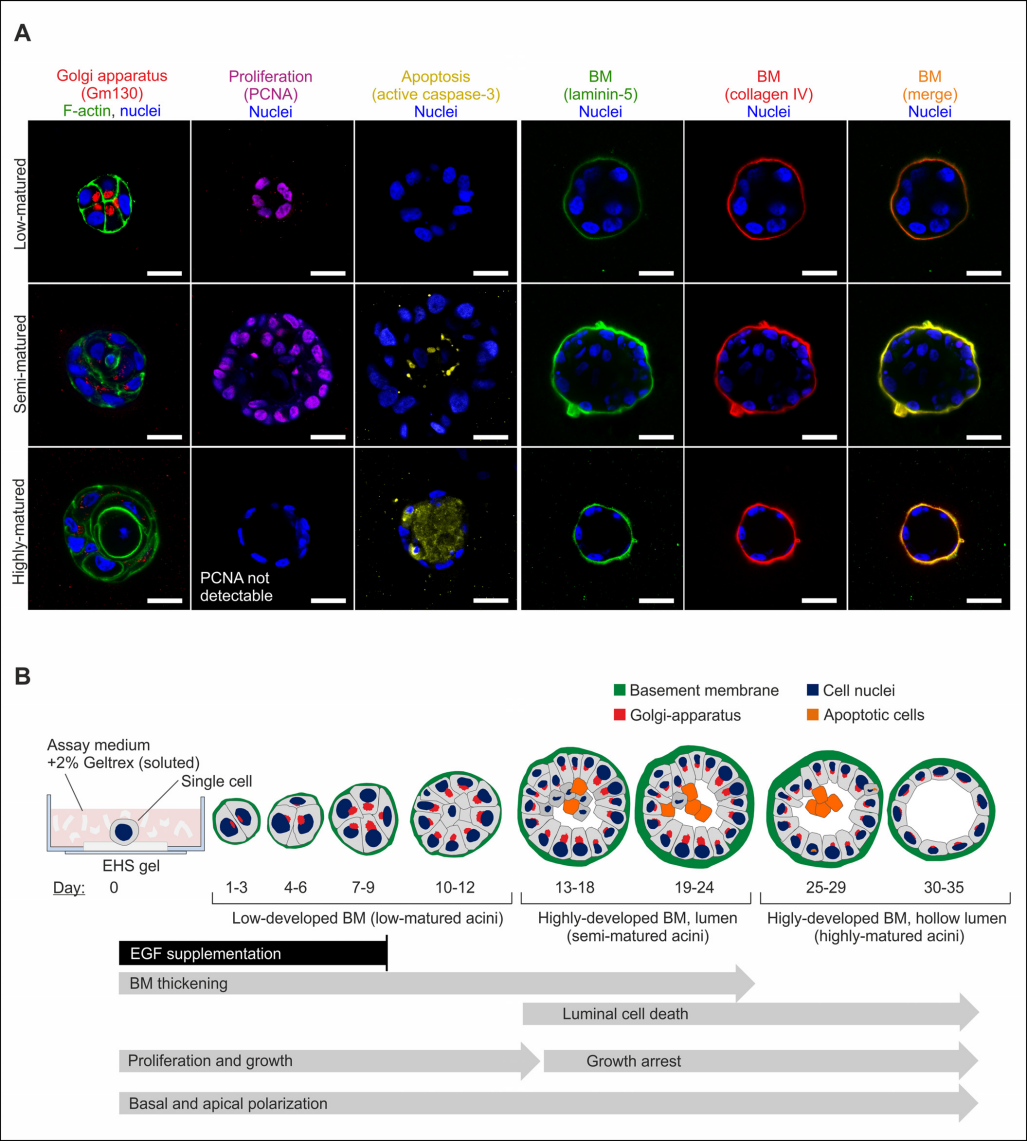
Capturing the differentiation process of MCF10A acini. **A.** Equatorial cross sections through MCF10A acini demonstrate the differentiation process leading to apical and basal polarization. Basal polarization markers: laminin-5, collagen IV (BM formation). Apical polarization markers: GM-130 (Golgi apparatus). Proliferating-nuclear-antigen PCNA and active caspase-3 marked s-phase and apoptotic cells, respectively. DRAQ5 was used to counterstain cell nuclei, cytoskeletal F-actin was stained with actin-phalloidin. Note that stains were performed on different spheres for low (1–12 days)-, semi (13–24 days)-, highly (25–35 days)-matured states. Scale bars = 20 μm. **B.** Immunofluorescence data were used to infer the development of acinar structures depending on temporal EGF withdrawal. Temporal progression is highlighted with arrows. Acini were grouped according to their differential grades.

Next, we tested for potential effects of our newly introduced EGF protocol on the whole acinar differentiation schedule, including BM development. 3D cultures were grown up to 33 days and fixed at distinct time points to conserve respective maturation stages. Well-established acinar polarization and proliferation markers [17] were used to visualize progressive differentiation from single cells to multicellular acini (Fig 2A). From these data, a generalized time line of MCF10A acinar maturation with active contribution of EGF was established (Fig 2B). Based on the chronological sequence of BM development and hollow lumen formation, acini were categorized as low-, semi-, and highly-matured. In detail, apical polarization (luminal Golgi orientation) became evident during the first 7 days. Basal polarization started instantaneously on day 1 by deposition of major BM components. BM thickening peaked after 13 to 24 days (semi-matured acini group) resulting in basally and apically polarized acini with ellipsoidal shape and highly-developed BM scaffolds. Longer cultivation periods did not lead to a detectable BM maturation or thickening. Cell proliferation proceeded even after EGF-depletion for approx. 5 days, as indicated by strong PCNA stain and resulted in increased acini sizes (Fig 2A). Luminal clearance started after 13 days with initially sparse apoptosis events and peaked after 30 days. Here, single layered acini with hollow lumen were eventually formed (highly-matured acini group). Please note that the following biophysical analyses were focused on low- and semi-matured acini due to their divergent BM developmental stages (Fig 2B).

### The basement membrane regulates substrate permeation in breast acini

**Fig 3:**
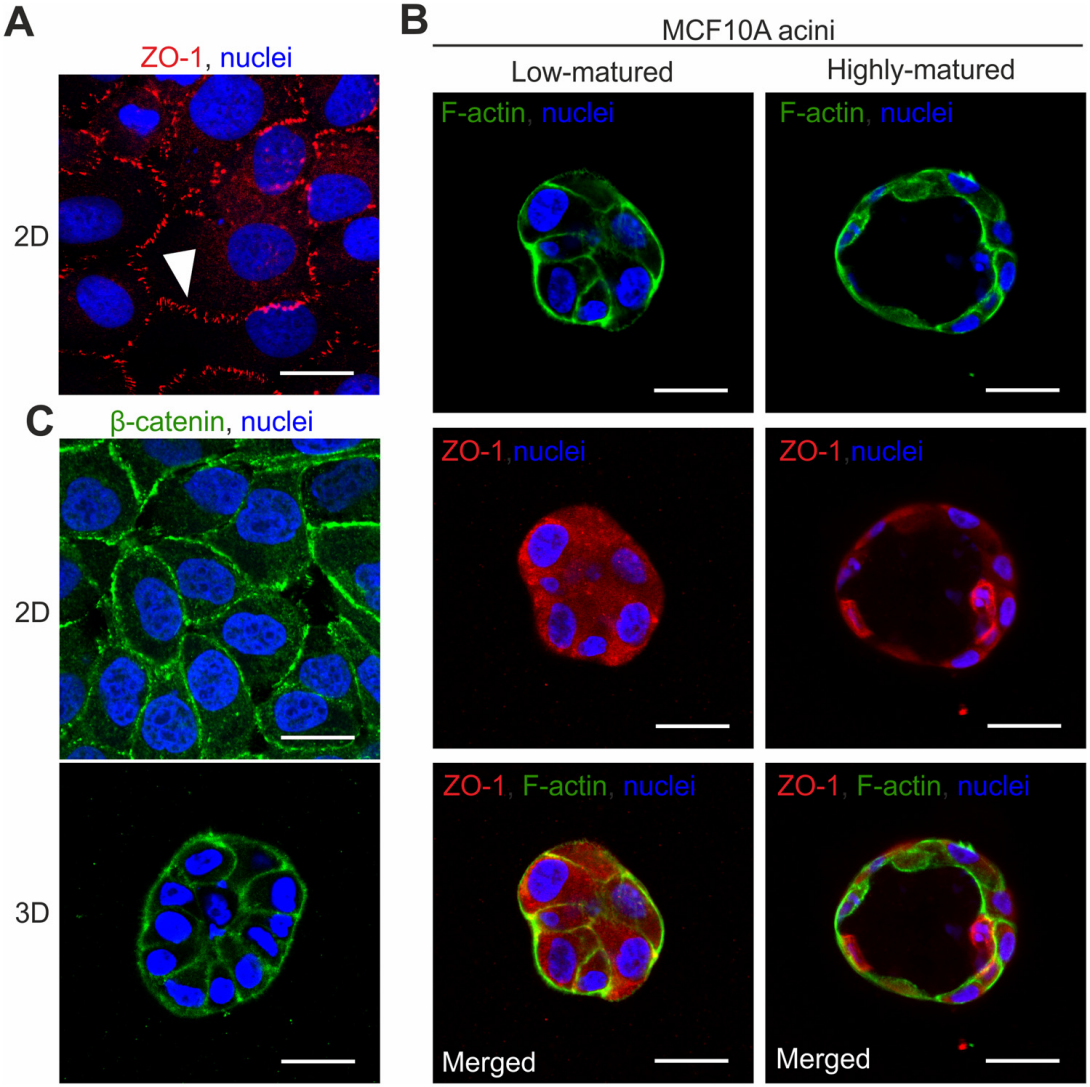
Tight junction formation in MCF10A cells depends on culture conditions. **A.** The tight junction (TJ) protein ZO-1 is visible as characteristic kissing points in 2D MCF10A monolayer cluster (white arrow head). **B.** Representative immunofluorescence stains for low- and highly-matured acini. Cortical F-actin signals (green) indicate acinar cell boundaries. ZO-1 protein (red) was only present as diffuse signal within the cytoplasm, irrespective of acinar-maturation states. The merged ZO-1 and F-actin stains demonstrate loss of colocalization and the absence of TJ-complexes. **C.** β-catenin signals indicate homogeneously distributed cell-cell contacts (adherence junctions) in both 2D MCF10A monolayer and in 3D MCF10A acini. Scale bars = 20 μm.

To address the question of whether the BM could act as putative regulator of substrate permeation in the human mammary gland, we first tested MCF10A cells for their ability to form tight junction (TJ) complexes that could contribute to this process. MCF10A monolayer cell cluster form kissing points [19] that indicate a continuous TJ complexion (Fig 3A). In contrast, under our newly established 3D EHS-culture conditions no apical localization of TJ formation could be observed. Only diffuse cytoplasmic ZO-1 protein signals were detectable irrespective of acinar maturation states (Fig 3B). Notably, adherence junctions (AJ) were present in both MCF10A monolayers and acini, indicated by a continuous β-catenin distribution at cell-cell contact sites (Fig 3C). This result shows the principle formation of cell-cell contacts in MCF10A acini. Due to the lack of functional TJs, MCF10A acini are an ideal model system for an unbiased analysis of the BMs’ contribution to passive substrate influx.

**Fig 4:**
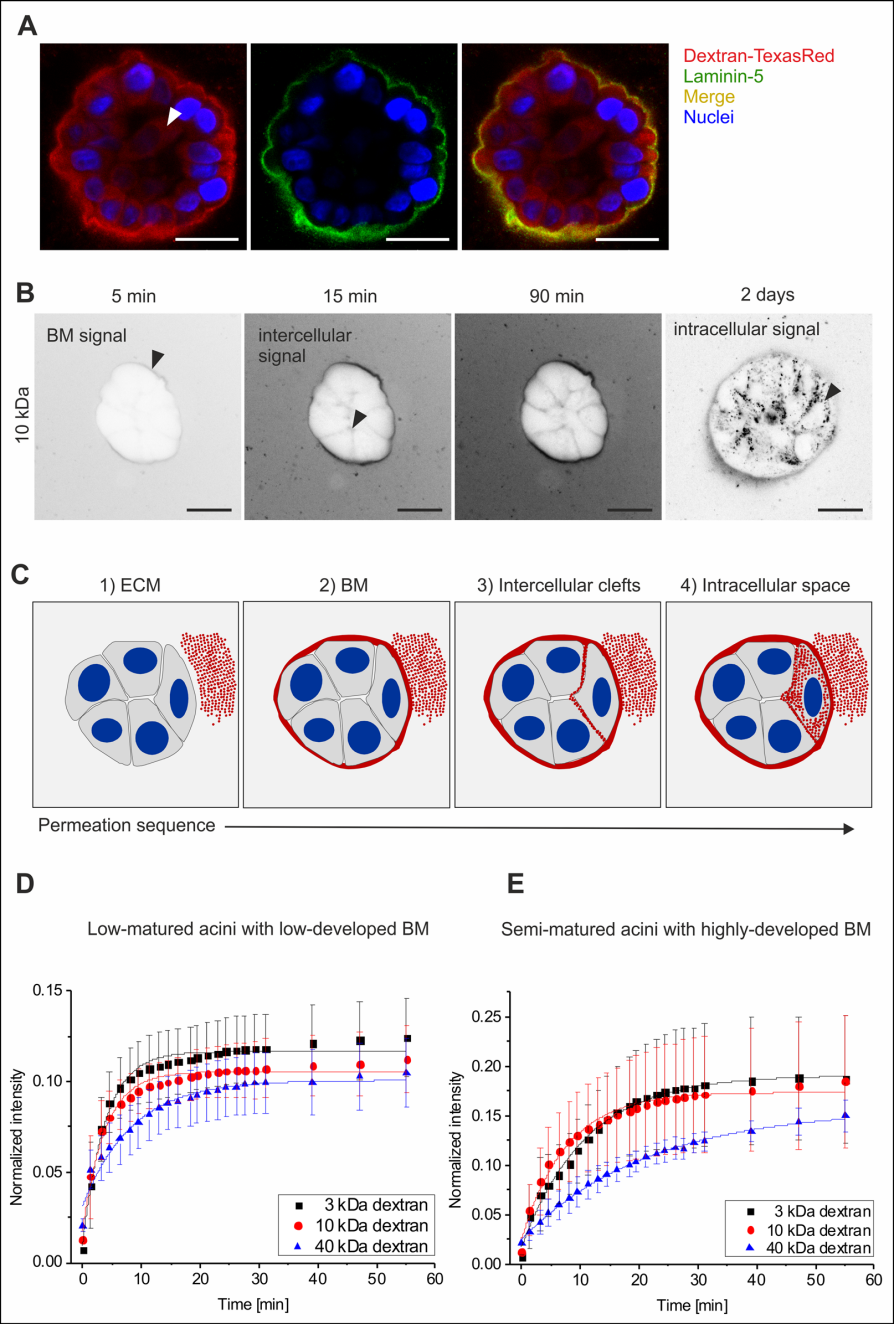
The basement membrane retards molecule influx into MCF10A acini. **A.** Colocalization of the BM specific laminin-5 protein with dextran molecules demonstrated the accumulation of dextran within the BM scaffold. The white arrowhead indicates a cytoplasmic localization (after 2 days) of dextran tracer (40 kDa). **B.** Representative image sequence of *in situ* dextran permeation in MCF10A acini with highly-developed BM (EHS-matrix embedded). Black arrows indicate first temporal appearance and spatial localization of dextran (10 kDa) (contrast inverted). **C.** Principal time course of molecule influx through the BM into MCF10A acini. Red: dextran, grey cytoplasm, blue: nuclei. Scale bars = 20 μm. Plots illustrate a semi-quantitative analysis of dextran permeation. Average fluorescence intensity profiles are shown for **(D.)** acini with low-developed BM and with **(E.)** highly-developed BM. Data are normalized to the background plateau for each molecular weight (see S2 Fig). Mean values with s.d. are shown. Full lines represent fits to a semi-empirical law (see S1 Protocol). Please note that absolute signal intensities of low- and semi-matured acini are not directly comparable because increased autofluorescence imposed the use of an adapted masking algorithm for the semi-matured sample group.

Fluorescent (TexasRed-labelled) dextran-tracers of increasing size (3 kDa, 10 kDa and 40 kDa) were used to systematically analyse diffusion within MCF10A acini with low- and highly-developed BM scaffolds using low-, and semi-matured groups, respectively (see Fig 2B). The analysis of highly-matured acini was not feasible since apoptosis caused autofluorescence artefacts (see supporting information, S1 Fig). After the addition of dextran substrates to EHS-embedded acini, a halo-shaped dextran signal appeared around the acini. Positive laminin-5 colocalization demonstrated that this accumulation was BM scaffold localized (Fig 4A). Over time the dextran signal became evident within intercellular cleft regions, and finally localized inside the cytoplasm of luminal cells (Fig 4B). This first qualitative analysis of dextran diffusion clearly indicated an increasing molecule retardation effect that correlated positively with dextran size and, more importantly, with proceeding BM development. Based on all data, a generalized molecule diffusion sequence through the BM was constructed (Fig 4C). Qualitative analyses of EHS-embedded acini (low- and highly-developed BMs) resulted in comparable effects (S1 Fig).

Next, we aimed to quantify the observed BM’s substrate retardation effect. To this end acini had to be analyzed without a surrounding ECM because the additional retardation introduced by the EHS-matrix substantially complicates data evaluation (see Fig 4B). Acini were removed from the EHS matrix, fluorescent dextran was added and the resulting process was recorded. To analyze the permeation process, a masking algorithm was developed that basically identifies the exterior space, the BM boundary itself and an acini interior. The resulting fluorescence intensity values over the whole masked domain (acini interior) were averaged for every time point. Representative intensity profiles normalized with the background signals (ROIs from exterior space) are shown in Fig 4D and 4E. Given the impossibility to determine the acinar enclosed volume and the BM surface, it was not possible to obtain the exact permeability coefficient of the BM. By fitting the profiles with a simple semi-empirical law (S1 Protocol), the characteristic time constant for the fluorescence intensity increase in the acini interior (τ) and in ROIs the exterior background (*τ*_0_) is extracted. (*τ*_0_ describes the speed of free diffusion and mixing of the dextran solution on the imaged focal plane). The time constant *τ* characterizes the combined retardation effect of BM permeation barrier and free diffusion. By computing the difference Δ*τ* = *τ* − *τ*_0_, a semi-quantitative comparison between the permeability speed of tracer sizes through low- and highly-developed BM scaffolds is described (for detailed technical information see S1 Protocol).

This approach clearly indicated that low-developed BM scaffolds (low-matured acini group) do not act as a permeation barrier. Solutes of the lowest molecular weights (3 and 10 kDa) permeated through the membrane almost as fast as they freely diffuse in solution, as indicated by very low values of Δτ (Δτ_3kDa_ = 0.7 min; Δτ_10kDa_ = 0.8 min). A slight retardation effect was only observed in the case of the largest dextrans (Δτ _40kDa_ = 1.8 min, p < 0.001) (Fig 4D). On the contrary, acini with highly-developed BMs (semi-matured acini group) proved to act as clear size-dependent permeation barriers: the considerably higher retardation grew with the dextran size (Δτ _3kDa_ = 3.1 min; Δτ _10kDa_ = 4.1 min) and peaked for the 40 kDa molecules (Δτ _40kDa_ = 12 min, p < 0.001) (Fig 4D). Taken together, this approach clearly validated a significant increase in molecule retardation with progressing BM development, which was further supported by our preliminary qualitative results.

**Fig 5:**
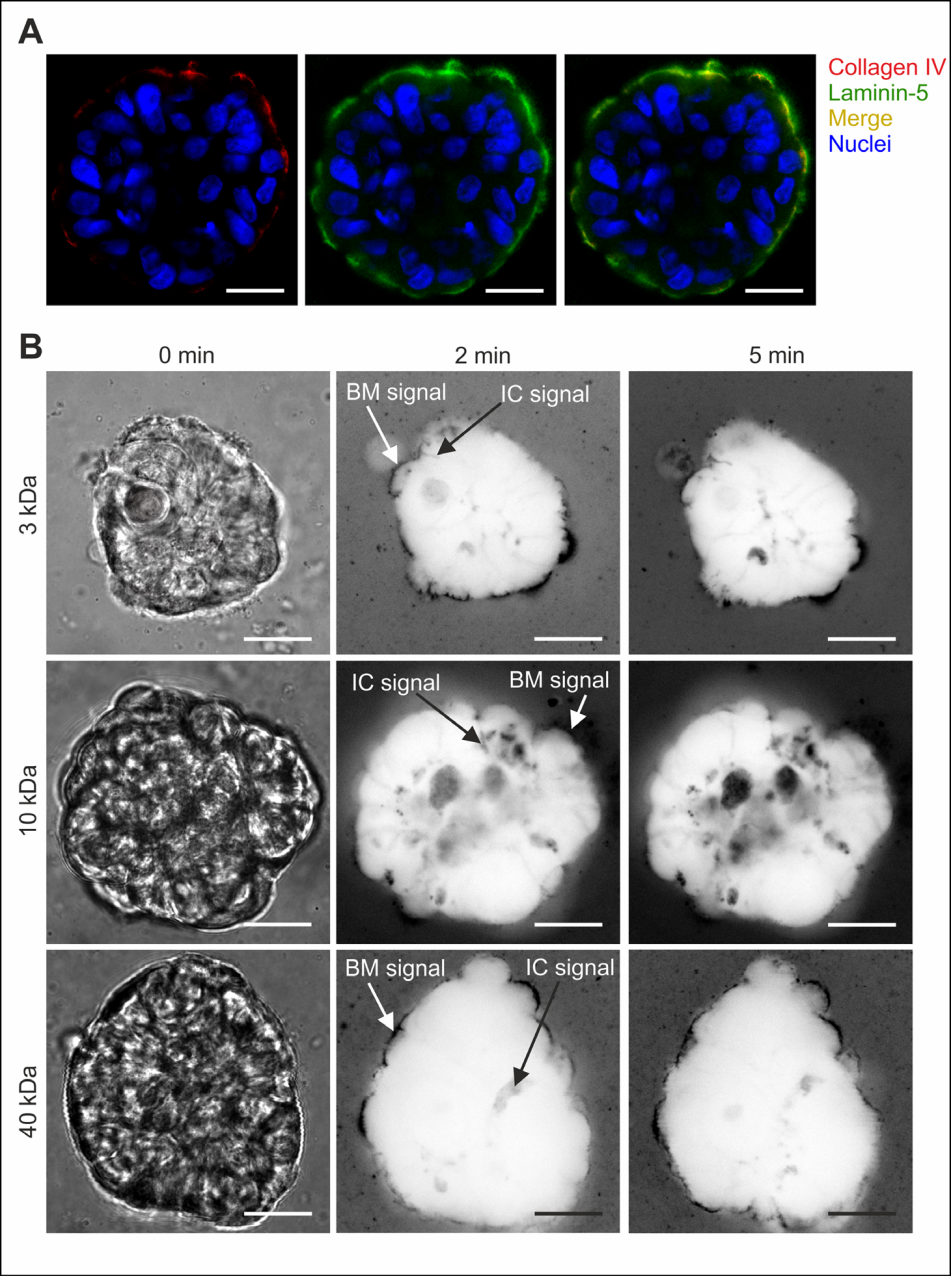
Intact collagen IV meshwork is essential for size-dependent molecule retardation. **A.** Control stain of BM specific collagen IV protein after enzymatic collagenase IV treatment. Comparative detection of laminin-5 protein demonstrated the colocalization of residual collagen IV protein within the remaining BM structure. **B.** The time-lapse of dextran influx in collagenase IV treated MCF10A acini with highly-developed BM (semi-matured group) is independent of the dextran tracer size. Left panel (0 min): bright field images. Other panels: representative dextran signal appearance (contrast inverted images) at indicated locations was independent from dextran molecular weights. IC: Intercellular cleft signal. Scale bars = 20 μm.

In order to investigate whether the observed molecule retardation effects correlate with an intact BM scaffold, we finally addressed the collagen IV meshwork as its major structural component. Semi-matured MCF10A acini were treated with collagenase IV to selectively disrupt their collagen IV meshwork. Interestingly, selective fragmentation of collagen IV additionally resulted in inhomogeneous laminin-5 staining suggesting for collagen IV as main structure-sustaining component of BMs (Fig 5A). Subsequent permeation measurements demonstrated that the BM-specific dextran signal did not longer appear as the halo-like structure typically found in native acini, but rather looked fragmentary and diffuse (Fig 5B). In contrast to native acini with intact BM structures (see Fig 4B), no size-dependent dextran influx retardation was evident in collagen IV-free acini (Fig 5B). In detail, dextran signals were visible in the BM and in acini interior (intercellular clefts) after 2 minutes—notably independent of the dextran size (Fig 5B).

### The basement membrane contributes to acinar mechanical integrity

**Fig 6:**
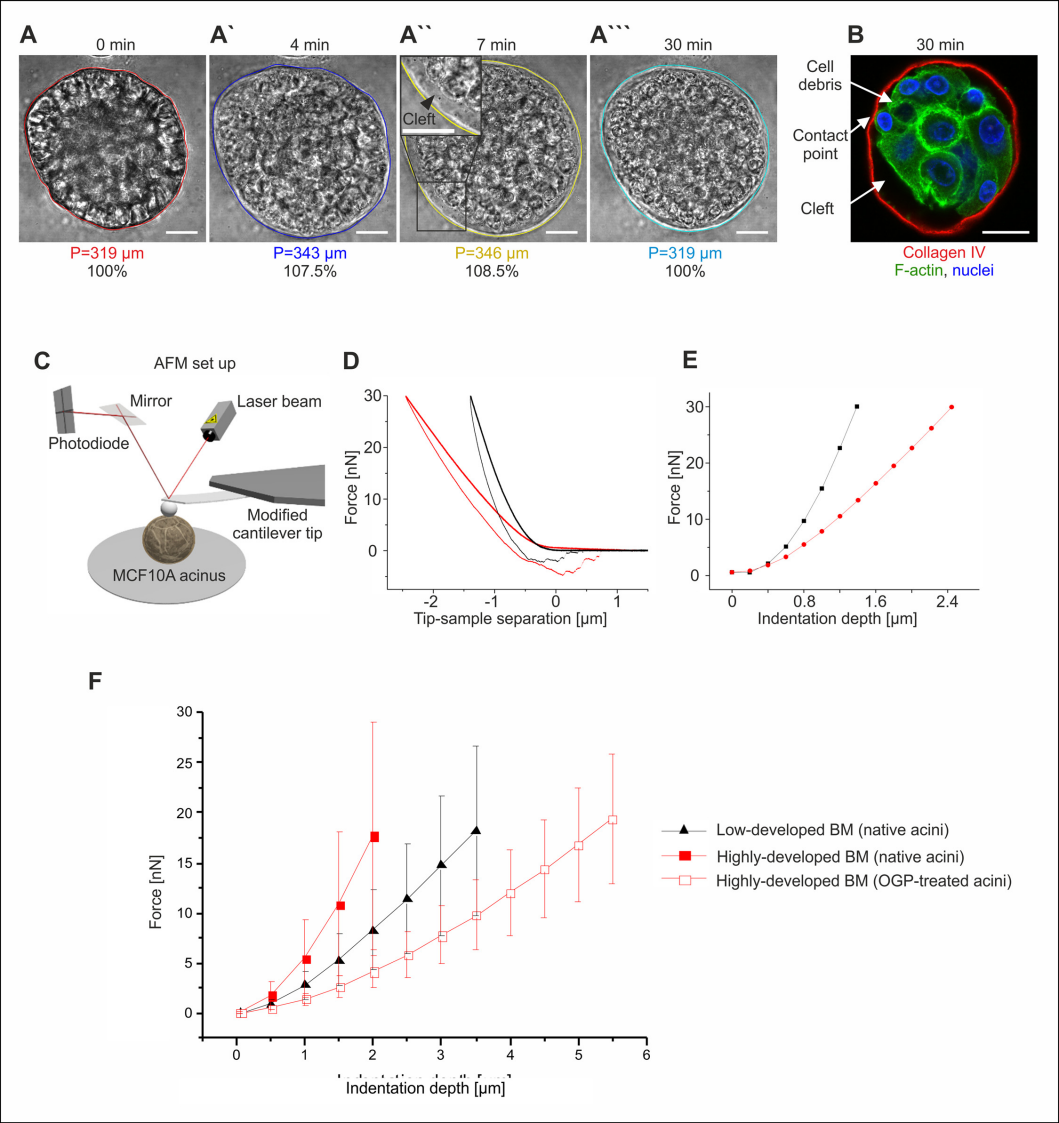
The basement membrane sustains the mechanical integrity of breast acini. The basement membrane sustains structural integrity without cellular networks. **A-A”.** Representative MCF10A acini embedded in EHS matrix were treated with OGP detergent. Acinar swelling and cell-BM separation are indicated by newly formed cell-free clefts during the first seven minutes (see inset). **A”‘.** Finally, acinar structures shrank to initial size. **B.** Only sparse contact points were visible between BM (red) and cell debris (green) after OGP-treatment. Nuclei (blue) were counterstained with DRAQ5. Scale bars = 20 μm. **C.** Schematics of the experimental AFM set up. **D.** AFM approach-retraction cycles as performed on the same 17-days old MCF10A acinus before (black) and after (red) OGP-treatment. **E.** Force values needed to reach a certain indentation depth into the acinus as directly read from the raw data in plot B. **F.** Plot illustrates the force distributions for increased indentation depths in native acini with low-developed BM (n = 40), native acini with highly-developed BM (n = 40) and OGP-treated acini with highly-developed BM (n = 31). Mean values with s.d. are shown. Differences between native and OGP-treated samples are highly significant (p < 0.001).

We addressed the question of whether the BM structure contributes to the mechanical stability of the human breast gland. To isolate the contribution of the BM to the mechanical integrity of breast acini, cell-cell contacts and cell-matrix anchorage had to be destroyed. To this end the detergent octyl-β-D-glucopyranoside (OGP) was used in order to efficiently disrupt the lipid bilayer of cell membranes while leaving BM protein scaffolds unaffected [20]. The first observation after OGP application was an overall swelling of MCF10A acini that was most likely caused by a transient increase in osmotic pressure due to cellular breakdown and the release of osmotically active material within the BM shell. After 7 minutes, OGP-treated acini (n = 21) showed highly significantly (p < 0.0001) increased acinar perimeters (P) (mean P = 227 μm, s.d. = 59 μm, Fig 6A”), when compared to the matched untreated sample (mean P = 195 μm, s.d. = 54 μm, Fig 6A). This swelling was reversed within 30 minutes and accompanied by cellular disorganization (Fig 6A to 6A”‘). In contrast, the BM retained structural integrity as indicated by halo-shaped collagen IV detection. Debris, residual cell nuclei and diffuse F-actin remained inside the acinar structure (Fig 6B). Finally, OGP treatment resulted in cleft formation due to contact loss between BM and cell membranes (Fig 6A” and 6B).

After having established a substantial separation of the BM from the acinar cell cluster, the effect of cells on the mechanical properties of the acinar BM was investigated by means of AFM (Fig 6C). Both acini with low-and highly-developed BM (low- and semi- matured groups) were mechanically indented before and after OGP treatment. For each sample, multiple indentation cycles were performed and the accurate overlap of the raw data curves indicated the overall elastic behavior of native acini (see S3 Fig). For the semi-matured acini, showing a highly-developed BM shell, AFM indentation after OGP treatment only rarely led to a partial plastic deformation. In the case of the low-developed BM, though, the osmotic swelling induced by OGP treatment was sufficient to cause a BM fracture for nearly every acinus analyzed. This already demonstrated that partially developed BMs offer a much lower mechanical resistance against external loads than well-matured ones. Additionally, fractured BMs exhibited higher unspecific adhesion to the AFM indenter tip and hindered a reliable analysis. A representative single AFM cycle performed on the same semi-matured acinus before and after OGP treatment is given in Fig 6D. The clear difference observed between the curves already points at a softening of the sphere after detergent incubation. The forces needed to reach a certain indentation depth in the acini can be directly read from the indentation curves (Fig 6E). An overall comparison revealed a highly significant (p < 0.001) softening of the decellularized BM shell population: native acini expressing a fully matured BM required about 3.9-fold higher indentation forces (5.5 nN, s.d. = 3.8 nN, n = 40) to reach an indentation of 1 μm compared to decellularized samples (1.4 nN, s.d. = 0.6 nN, n = 31). The resistance effect became even more pronounced at higher indentation depths (Fig 6F). Nevertheless, even after decellularization, the mechanical integrity of the BM shell was retained upon AFM indentation performed at loads as high as 20 nN (Fig 6F). Due to the defined geometry of the used AFM indenter glass sphere (radius: 22 μm) and a given indentation depth the corresponding force can be approximately converted in pressure. As a result, the force of 20 nN at 2 μm indentation depth, equates to an average applied compressive pressure of approximately 80 Pa. Low-matured MCF10A spheres systematically required less force or pressure to be indented to the same depth (1.5 folds less for 1 μm) thereby proving to be softer than acini with highly-developed BM shells.

## Discussion

The biophysical blueprint of breast gland tissue is still poorly understood. This study therefore aimed to characterize the BM as a biophysically active key unit in breast acini. To our best knowledge, we show for the first time that EGF withdrawal after 9 days enhances the frequency of MCF10A acini maturation significantly with reliable BM formation and six-fold increased lumen formation. This novel finding is in line with an analogous EGF depletion after 7 days, which optimized the differentiation of the comparable HMT-3522-S1 human breast acini model [21]. Such significant impact of EGF-withdrawal on morphology has been shown to foster interdigitating desmosome formation due to actin remodeling in both MCF10A and HMT-3522-S1 monolayer. Moreover, those interdigitating structures were functionally linked to enhanced mechanical cell-cluster stability [22]. Of note, even after EGF depletion MCF10A acini continued to grow for several days, which could be related to the suggested growth factor reservoir and delivery function of BM scaffolds [21]. Additionally, the less powerful stimulation by cholera toxin might also contribute to a prolonged growth [3,23].

Luminal clearance is the final step of breast acini differentiation and requires apoptosis [24,25]. Matrix-adhesion loss of the inner cell mass leads to EGFR down regulation, pro-apoptotic BIM protein expression and finally anoikis induction. Interestingly, EGF withdrawal causes BIM upregulation and anoikis in detached MCF10A cell suspension [26]. A limited EGF stimulation for 9 days could hence be mechanistically related to such BIM-dependent anoikis stimulation and foster lumen clearance in MCF10A acini. However, lumen clearance seemed temporally shifted a few days when directly compared to a well-established and acknowledged work [17]. A plausible explanation for this shift could be the delayed apoptotic response upon anoikis stimulation in this cell type of up to 3 days [27]. Nevertheless, our MCF10A acini feature all morphological hallmarks and average size typically achievable for this *in vitro* model [16]. Thus, our method of limited EGF-stimulation should be seen as a potential addition to existing and still highly valuable protocols [21,28], especially when undesirable differentiation heterogeneities occur.

For biophysical BM analyses, we categorized acini for low- and highly-developed BMs, to ensure experimental reproducibility and to ease data interpretation. Of note, the analysis of acini featuring a highly-developed BM and a hollow lumen has been purposely omitted, since the contribution of lumen formation to BM mechanics was not analyzed and further BM maturation between semi-, and highly-matured acini could not be detected. Furthermore, frequent apoptotic autofluorescence artifacts within highly-matured samples would have impeded a reliable quantification of dextran permeation.

We hypothesized that the BM scaffold contributes to molecule permeation and influences normal and malignant breast gland phenotypes vitally. For an unbiased analysis of BM-dependent passive molecule transport we verified the lack of TJ complexes in our MCF10A acini model, which is in line with previous observations [16]. Functional TJ complexes would distort the role of the BM for passive diffusion of ions and small water-soluble molecules into the intercellular space and lumen [29]. First dextran tracer experiments were performed under physiological conditions, meaning that acini were analyzed within the EHS-matrix. Intriguingly, we found a general molecule retardation effect of the BM scaffold that depended on dextran size and also BM development stage. Moreover, the osmotic influx-driven swelling and shrinking of decellularized BM shells underlined the BM function as molecule sieve in breast gland tissue. In order to verify these initial findings we determined the molecule permeations speed for BMs of low-, and semi-matured breast acini.

Our data demonstrate the functional relation between a proceeding acinar maturation and a size-dependent permeation barrier function of the BM scaffold. A significant delay of molecule permeation was exclusively evident in semi-matured acini due to highly-developed BM scaffolds that were thicker than the low-developed ones. Unfortunately, collagen IV stains only allowed visualizing a relative BM enlargement in semi-matured acini. In order to quantify the BM thickening progress, the topography of isolated, flat scaffold layers would have to be analyzed by ultra-sensitive AFM imaging. This would allow correlating BM scaffold thickness and diffusion time constants. Additionally, the molecule diffusion speed depended also on the BM pore-size, as large dextrans (40 kDa) displayed a characteristic and significantly higher permeation time constant compared to the small tracers. The dependence of solute permeation on BM thickness and pore size is expected from investigations on hydrogels and technical membranes where an inverse dependence of permeability on film thickness and an even stronger decay with decreasing pores size has been found [30,31].

40 kDa Dextran features a hydrodynamic diameter of approx. 9 nm [10]. Of note, our TexasRed labeled tracer might have a slightly altered hydrodynamic diameter. Since these molecules were capable to pass the BM barrier, we suggest an estimated BM pore fraction of at least 9 nm in diameter, which contributes to the size-dependent molecule retardation. Compared to breast BMs the average pore size of corneal epithelial BMs appear clearly larger (32–112 nm) [32], indicating that the pore size might be related to the organ function. So far, pore sizes for the BM of human breast glands have not been described and comparable filtration effects have been shown only for glomerular BMs whose main permeability barrier lies in the range of 40 to 200 kDa [10,33]. With regard to the fine-tuned control of breast gland differentiation, the lactogenic hormone prolactin is a good example for a small signaling factor that has to cross the BM barrier to fulfill its biological function. The biologically active form of prolactin is about 23 kDa with an estimated electrostatic diameter of 80 Å [34]. Prolactin is produced within the pituitary gland and stimulates breast cell differentiation and lactation [35]. It can therefore be assumed that the BM pore span is at least 8 nm, which is in good agreement with our experimental result of 9 nm. Molecule size and the related diffusion properties are crucial parameters for drug design. Interestingly, polyglumex (38 kDa) a 45 times larger polymer-conjugated variant of the classical chemotherapeutic paclitaxel, has been described to be more permeable in tumor neovasculature than in normal tissue. Due to different tissue paucities, this results in 100-fold higher intratumoral drug concentrations, increased retention times, higher efficiency and less side effects on normal tissues [36]. Hence, the recommended molecular cancer drug size is ≥ 50 kDa, based on an enhanced molecule permeation and retardation in tumorous vasculature and lymphatic vessels [37]. This is in line with our finding for the increased retardation of 40 kDa dextrans through the BM of MCF10A breast acini. With regard to possible implications for breast cancer chemotherapy, such BM-mediated drug retardation could protect healthy glandular tissue from adverse cytotoxic effects, while BM-lacking tumor tissue would be targeted more efficiently. The BM could therefore be a crucial gatekeeper for molecule diffusion, not only in terms of normal glandular development and differentiation, but also for chemotherapeutic drug delivery.

Interestingly, we found that a collagen IV meshwork disruption in the BM completely abolished the size-dependent molecule retardation effect. This result underlines the pivotal role of a native collagen IV deposition and assembling for the BM functionality as molecule diffusion barrier. The collagen chain composition changes during BM development [38] and is adjusted according to physiological requirements [8,39]. The increasing degree of intermolecular cross-linking of collagen IV meshes creates highly-specialized BM structures [39–41]. Collagen α3α4α5 chains have been postulated to be associated with increased macromolecule size-selectivity of kidney BMs [42]. Of note, comparable chains are located in mammary gland BMs [40,41] and might have a similar effect on molecule permeation. Additionally, ECM collagen IV scaffold organization triggers interstitial fluid pressure and regulates drug delivery in cancer therapy [43,44], which could also be important for molecule transportation through the BM. The collagen meshwork of breast gland ECM and of the BM is highly-dynamically remodeled by matrix metalloproteinases (MMPs) and mandatory for normal tissue and BM differentiation [5]. Furthermore, increased ECM stiffness induces MMP14 expression in MCF10A breast acini and leads to altered BM structures [45]. These findings indicate a close relationship between MMP activity and BM formation breast gland acini. Accordingly, an increased collagenase IV (MMP2, MMP9) activity has been linked to high tumor grades in breast cancer specimen and poor prognosis [46]. *In vivo*, MMP2 expression has been found in myoepithelial cells, which enclose the acinar cell layer and could therefore contribute to BM weakening and breakdown [47]. Such a BM breaching could alter the acinar influx of a multitude of secreted soluble factors and thus foster tumor progression. With regard to our MCF10A acini, it is most likely that dynamic MMP expression and secretion contribute to BM formation, composition and finally modulates its functionality as permeation barrier. We suggest that collagen IV composition could change during MCF10A acini maturation and might contribute to the increased substrate size selectivity of acini with highly-developed BM shells. Taken together, we suggest that BM permeation is modulated by scaffold thickening, collagen composition and organization, as well as resultant pore sizes. Further research is necessary to resolve the interplay of these mixed effect sizes and the putative MMP remodeling function in more detail.

In order to decipher the important role of the BM as mechanical shielding, we aimed to quantify the mechanical property of the BM for normal breast gland stability. Our simple acini decellularization experiment indicated for an elastic behavior of the BM shell. During acinar swelling and subsequent shrinking, cell debris detached from the still roundish and intact BM shells, reflecting a thorough loss of hemidesmosomal cell-BM connections [16]. From this we conclude that cell-BM contacts are not principally mandatory for BM stability.

Finally we quantified the actual force resistance of BM structures by AFM as major technique for the ultra-sensitive measurement of force-resistance against indentation of single cells [48] and multi-cellular clusters [49]. Although a possible influence of the acini isolation on the BM scaffold cannot be totally excluded, BMs of matrix-embedded and isolated acini appeared identical in terms of shape and collagen IV and laminin V protein content, according to the immunostainings. This observation mirrors the inherent robustness of the BM shell, when compared to the surrounding loose collagen gel matrix. Since AFM indentations depths went up to 5.5 μm and thus penetrated beyond the BM layer, it is unavoidable that force resistance values of native breast acini not only resulted from different BM developmental stages but also depended on material responses of cells and cell-cell contacts. Therefore, the force resistance of BM shells before and after decellularization were analyzed. The finding that native MCF10A acini required up to 3-fold higher forces to reach identical indentation depths, when compared to cell-free BM shells, underlines the natural complexity of MCF10A breast acini. Native acini, like breast gland tissue, are highly organized cell cluster with a hierarchal network of cytoskeletal components such as actin fibers, microtubules and intermediate filaments, and of cell-cell connections (mostly adherence junctions). Moreover, acinar cells anchor themselves to the shielding BM scaffold by hemidesmosomes and α6β4 integrin receptor bonds [16]. Hence, the higher force resistance of native acini results from structural contribution of local cell-cell and cell-BM adhesions. The fact that highly-developed, decellularized BM shells remained structurally intact upon repeated AFM indentation further proved their elastic nature and demonstrated the fundamental role played by the BM in conferring mechanical stability to human breast acini. Most importantly, cell-free BM shells were still capable to withstand loads of 20 nN demonstrating their inherent mechanical stability, which contributes to the overall strength of MCF10A breast acini. The maximum force resistance of 20 nN, equates to a compressive pressure of 83 Pa. Importantly, this value lies in a range described for human breast gland tissue with different pathological conditions and is therefore physiologically relevant. The interstitial fluid pressure in healthy breast gland tissue and other benign conditions lies in the range of 40 Pa to 53 Pa [50]. MCF10A breast acini are therefore capable of withstanding physiological loads that occur within normal conditions *in vivo*, underlining their relevance as biophysical *in vitro* model for breast gland development. In contrast, benign breast tumors and invasive ductal carcinomas exhibit ten- to hundredfold increased pressure values of 480 Pa to 3.9 kPa, respectively [50]. Thus, the compressive resistance we deciphered for isolated BMs *in vitro* resembles the natural condition of non-tumorous breast gland tissue *in vivo*. Moreover, it underlines the importance of the BM to maintain structural integrity by compensation of tissue pressure, which dramatically increases during cancer progression to an extend that exceeds the structural strength of the BM by orders of magnitude and finally might contribute to BM breakdown and cell invasion.

As discussed earlier for molecule diffusion, protein composition should be of paramount importance for BM mechanical stability. In fact, collagen IV has been already described to structurally sustain BMs of various organs [6,7]. The strength of human breast gland BMs strongly depends on density, fiber orientation, fiber thickness and α-chain composition of the collagen IV mesh [39–41]. To our knowledge, this is the first report that quantitatively defines the force resistance against mechanical compression of human breast gland BM scaffolds. Nevertheless, future investigations will have to clarify the actual interplay between molecular components, such as collagen IV, overall architecture and mechanical stiffness of the breast gland BM.

In conclusion, our data elucidate that the BM sustains significant mechanical support in breast gland acini and functions as passive diffusion barrier for macromolecules. To decipher such biophysical properties of breast gland tissue, it is vital to link the biomechanical phenotype with biochemical knowledge of breast cancer development and progression. Finally, this will open new perspectives for the identification of molecular drug targets and breast cancer therapy.

## Materials and Methods

### Cell maintenance

MCF10A cells were obtained from ATCC (Manassas, VA, USA) and maintained at 37°C in a humidified environment of 5% CO_2_ and 95% air. The medium composition was adopted from Debnath and co-workers [17]: DMEM/F12 (Life Technologies, Carlsbad, CA, USA) growth medium containing 5% horse serum (Life Technologies), 20 ng mL^-1^ EGF (Sigma-Aldrich, St. Louis, MO, USA), 0.5 μg mL^-1^ hydrocortisone, 100 ng mL^-1^ cholera-toxin, 10 μg mL^-1^ insulin, 100 U mL^-1^ penicillin and 100 μg mL^-1^ streptomycin (Sigma-Aldrich).

### Morphogenesis assay

MCF10A acini were cultured on growth factor reduced EHS-gel bed (Geltrex, Life Technologies) according to previous publications [17,25], with modification regarding growth factor supplementation. Assay media: DMEM/F12 (Life Technologies) containing 2% horse serum (Life Technologies), 5 ng mL^-1^ epidermal growth factor (day1 to 9) (EGF, Sigma-Aldrich), 0.5 μg mL^-1^ hydrocortisone (Sigma-Aldrich), 100 ng mL^-1^ cholera toxin (Sigma-Aldrich), 10 μg mL^-1^, 100 U mL^-1^ penicillin and 100 μg mL^-1^ streptomycin (Sigma-Aldrich). The assay medium was supplemented with Geltrex in a non-gel forming concentration (2%) and was changed every third day.

### Immunostaining and imaging

EHS-embedded MCF10A acini were washed with cytoskeleton-buffer (CB: 5 mM EGTA, 5 mM glucose, 10 mM MES, 5 mM MgCl_2_, 150 mM NaCl, 1 g L^-1^ streptomycin; Sigma-Aldrich) at RT and fixated with 2% formaldehyde (Sigma-Aldrich) and 0.5% glutaraldehyde (EM Grade, Polysciences, Inc., Warrington, PA, USA) in CB for 20 minutes (RT). After quenching with 1% NaBH4 (freshly prepared in CB) (Merck Millipore, Billerica, MA, USA), samples were washed twice (5 minutes each) with 30 mM glycine-CB at RT. Permeabilization was performed with 0.5% Triton X-100-CB (Sigma-Aldrich) solution for 20 minutes at RT, or with methanol at -20°C for 10 minutes (for detection of PCNA, Cell Signaling Technology). Unspecific antibody binding was blocked with (0.1% BSA, 0.2% Triton X-100, 0.05% Tween 20, in CB; Sigma-Aldrich) containing 10% goat serum (Sigma-Aldrich) or 5% skim milk powder (Sigma-Aldrich), and 1% goat F(ab’)_2_ anti-mouse IgG (Life Technologies) for 2 hours at RT. Primary antibodies anti-laminin-5 (Merck Millipore, Billerica, MA, USA), anti-type IV collagen (Abcam, Cambridge, England, UK), anti-β-catenin (Sigma-Aldrich), anti-cleaved caspase-3 (Cell Signaling Technology), anti-TJP1/ZO-1 (Sigma-Aldrich), anti-PCNA (Cell Signaling Technology, anti-GM-130 (BD Biosciences, San Jose, CA, USA) were diluted in 1% blocking buffer in CB and incubated overnight at 4°C. Secondary antibodies coupled with fluorescent dyes (Life Technologies) were diluted (1% blocking buffer in CB) and applied to the samples for 45 minutes at RT in darkness. For direct actin-phalloidin (Life Technologies) stain, samples were only permeabilized after fixation, and stained for 45 minutes at RT. After two washing steps with PBS, DRAQ5 (Cell Signaling) stain was applied for 10 minutes at RT. Samples were imaged using a Zeiss C-Apochromat water immersion objective lens (63x, NA = 1.15) on a confocal laser scanning microscope 710 (cLSM710) with ZEN2011 software (Carl Zeiss).

### Dextran permeability assay

For *in situ* assays, acinar samples were gently washed with PBS (5 minutes, 37°C) and incubated in EGF-free assay medium with TexasRed-conjugated dextrans (3 kDa 133 μg mL^-1^, 10 kDa and 40 kDa 200 μg mL^-1^, E_x_/E_m_ = 585/905 nm, Life Technologies). Dextran signal was recorded every 5 minutes for 3 hours using a Zeiss C-Apochromat water immersion objective lens (63x, NA = 1.15) and a cLSM710 (Carl Zeiss, Jena, Germany) equipped with a live cell imaging device (37°C and 5% CO_2_). For the semi-quantitative experiments acini were isolated from Geltrex and transferred onto a glass bottom dish: Samples were gently washed with ice-cold PBS and incubated on ice in assay medium (phenol red-free) for 1 hours to break down the EHS-matrix. Acini were gently centrifuged (150 g), the supernatant was removed and the acini were re-suspended in PBS. After three washing steps purified spheres were resuspended in small volume of assay medium, transferred onto Geltrex covered (8 μL/cm^2^) glass bottom dishes and let to adhere for 20 minutes. Medium was premixed with the respective dextran tracer before imaging was started. Dextran signal was recorded every 1.5 minutes for 30 minutes, and every 8 minutes for further 5 hours. At least15 acini were analyzed out of each sample in at least three independent experiments. For detailed technical information about the masking algorithm and the empirical permeation time constant calculation please see S1 Protocol.

### Collagenase IV assay

Acini were washed with Hank's Balanced Salt Solution (HBSS, Life Technologies) and treated with 290 U mL^-1^ collagenase IV (Worthington Biochemical Corporation, Lakewood, NJ, USA) in HBBS for 3 hours. Subsequently, dextran permeability assays were performed as described.

### Basement membrane decellularization assay

Acini were treated with octyl-β-D-glucopyranoside (OGP, Sigma-Aldrich). Live cell imaging started with addition of OGP solution (1% in PBS). Images were taken every minute for the first 30 minutes, and every 5 minutes during the following 5 hours. Reference images were taken prior to OGP treatment. Subsequently, acini were fixated for immunofluorescence imaging.

### AFM measurements

The high sensitivity of AFM measurements imposed the need to eliminate any residues of loose gel matrix would have significantly altered the indentation read out. Embedded MCF10A were separated from Geltrex according to a previous protocol [51] with slight modifications. Acini were washed with ice-cold PBS for 5 minutes and incubated for 50 minutes in ice-cold EHS substrate break down solution (CRS, BD Biosciences). Subsequently, acini were gently pipetted out of the EHS solution, resuspended in ice-cold PBS, centrifuged for 5 minutes at 150 g (4°C) and transferred on a Petri dishes for analysis. All samples were treated identically to preserve the comparability of measurements. Force spectroscopy AFM was performed in in HEPES-buffered assay medium using a Nanowizard Life Science version instrument (JPK, Berlin, Germany) equipped with an inverted optical microscope (Axiovert 200, Carl Zeiss) for sample observation. Indenters were prepared by mounting glass microspheres (nominal radius of 30–50 μm) (Polysciences, Inc.) to silicon AFM tipless cantilevers of nominal resonance frequency f_0_ = 7 kHz and nominal spring constant k = 0.04 N m^-1^ (Nanoworld Arrow TL1Au with Ti/Au back tip coating) using two component glue (UHU plus Endfest 300; UHU, Bühl/Baden, Germany). Cantilever calibration: thermal noise method. Measured spring constant: 0.029 N/m. The exact glass bead diameter was determined by light microscopy (r = 22.32 μm). After thermal equilibration, at least three force curves were recorded on each analyzed sample. Cantilever speed (v) = 2 μm/s. Force set point = 30 nN. Afterwards, native acini were subjected to the BM decellularization assay and measured again.

### Statistical analysis

Acini perimeters were compared using the two-tailed nonparametric Wilcoxon-matched pairs test (95% confidence interval). AFM force distributions and permeation time constant distributions were analysed by using the two-tailed nonparametric unpaired Wilcoxon signed-rank test (95% confidence interval). All analyses were performed with Origin (9.0) software (OriginLab Corporation, Northampton, MA USA), R (2.155.3) and MatLab (R2015a) software (The MathWorks, Inc., Natick, MA USA).

## Supporting Information

S1 Fig Dextran influx in dependence of dextran size and MCF10A acini maturation. Time-lapse images demonstrate representative temporal and spatial dextran permeation in **(A.)** highly-, **(B.)** semi- and **(C.)** low-matured MCF10A acini. Arrow heads indicate first signal localization (contrast inverted). It has to be taken into account that intracellular signal interpretation was partially compromised in highly matured samples due to increased autofluorescence, which was caused by massive apoptotic events. BM: Basement membrane. Scale bars = 20 μm.
(TIF)

S2 Fig The basement membrane retards molecule influx into MCF10A acini. Average fluorescence intensity profiles recorded in **(A.)** low-matured acini and **(B.)** corresponding background ROIs and **(C.)** semi-matured acini and **(D.)** their background ROIs. Normalizations are with respect to the background plateau for each molecular weight. Bars indicate s.d. The superimposed curves are fits of S Eq. 1. For details of region identification see S1 Protocol.
(TIF)

S3 Fig Reproducibility of AFM indentations on MCF10A acini. The plot shows three consecutive AFM indentation cycles that were performed on a native MCF-10A acinus. The almost perfect overlap of successive curves indicated the absence of plastic deformation and an overall elastic behavior of the acinar structures under this deformation.
(TIF)

S1 Protocol Information about the masking algorithm and permeation data analysis. (PDF)

S1 Table Excel file with numerical data for graphs in Figs 1C, 1D, 4D, 6A, 6E and 6F. (XLSX)

## Abbreviations

AFM: Atomic force microscopy
BM: Basement membrane
EHS: Engelbreth Holm Swarm
EGF: Epidermal growth factor
MMP2: Matrix metalloproteinase 2
MMP9: Matrix metalloproteinase 9
OGP: octyl-β-D-glucopyranoside
P: perimeter
ROI: Region of interest
TJ: Tight junction
