# Supplementary figures and images for: The Acinar Cage: Basement Membranes Determine Molecule Exchange and Mechanical Stability of Human Breast Cell Acini
Source: PLoS One. 2015 Dec 16;10(12):e0145174. doi: 10.1371/journal.pone.0145174 (PMC4684506; doi:10.1371/journal.pone.0145174)

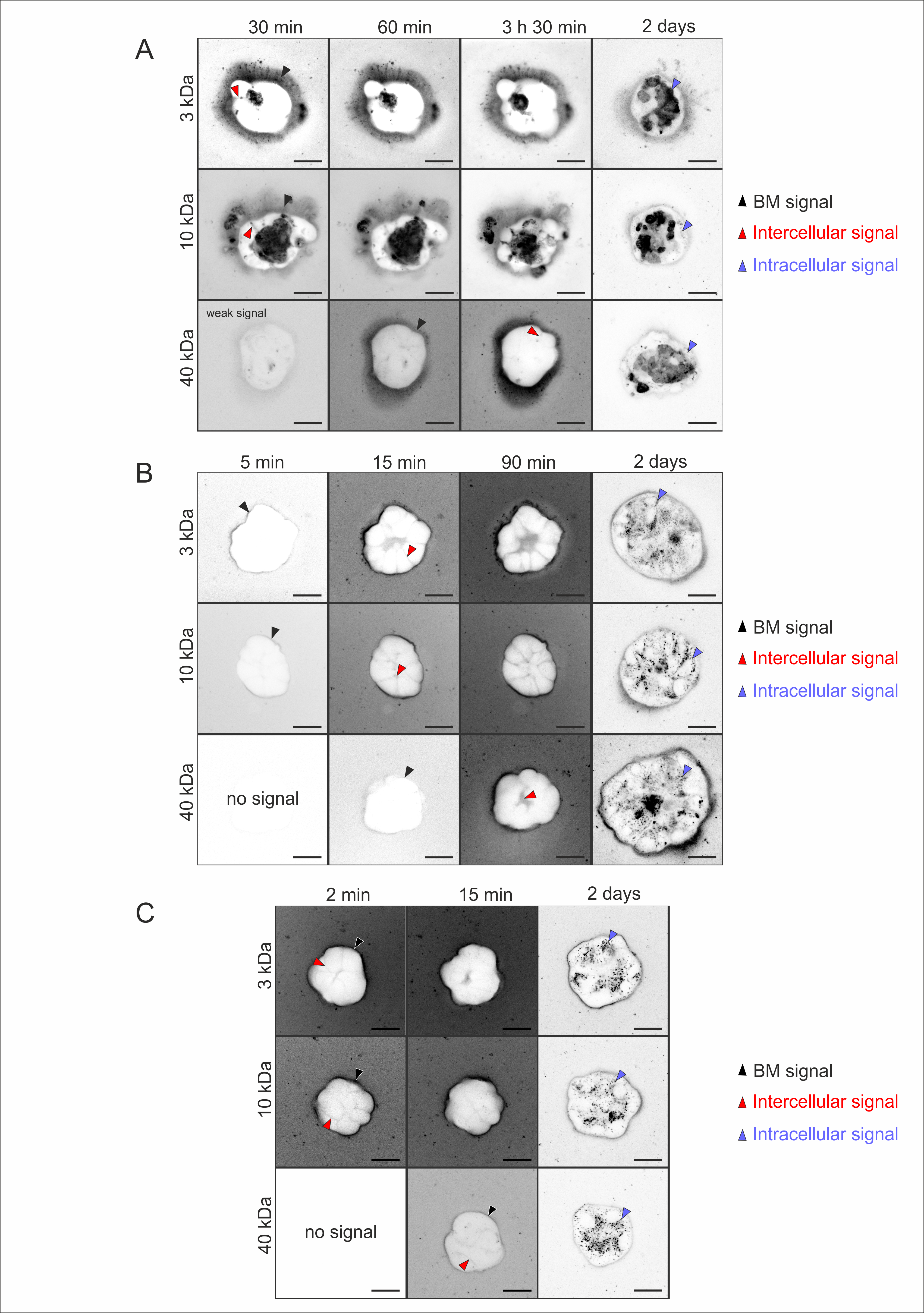

Supplement: S1 Fig — Time-lapse images demonstrate representative temporal and spatial dextran permeation in (A.) highly-, (B.) semi- and (C.) low-matured MCF10A acini. Arrow heads indicate first signal localization (contrast inverted). It has to be taken into account that intracellular signal interpretation was partially compromised in highly matured samples due to increased autofluorescence, which was caused by massive apoptotic events. BM: Basement membrane. Scale bars = 20 μm. (TIF) [file pone.0145174.s001.tif]

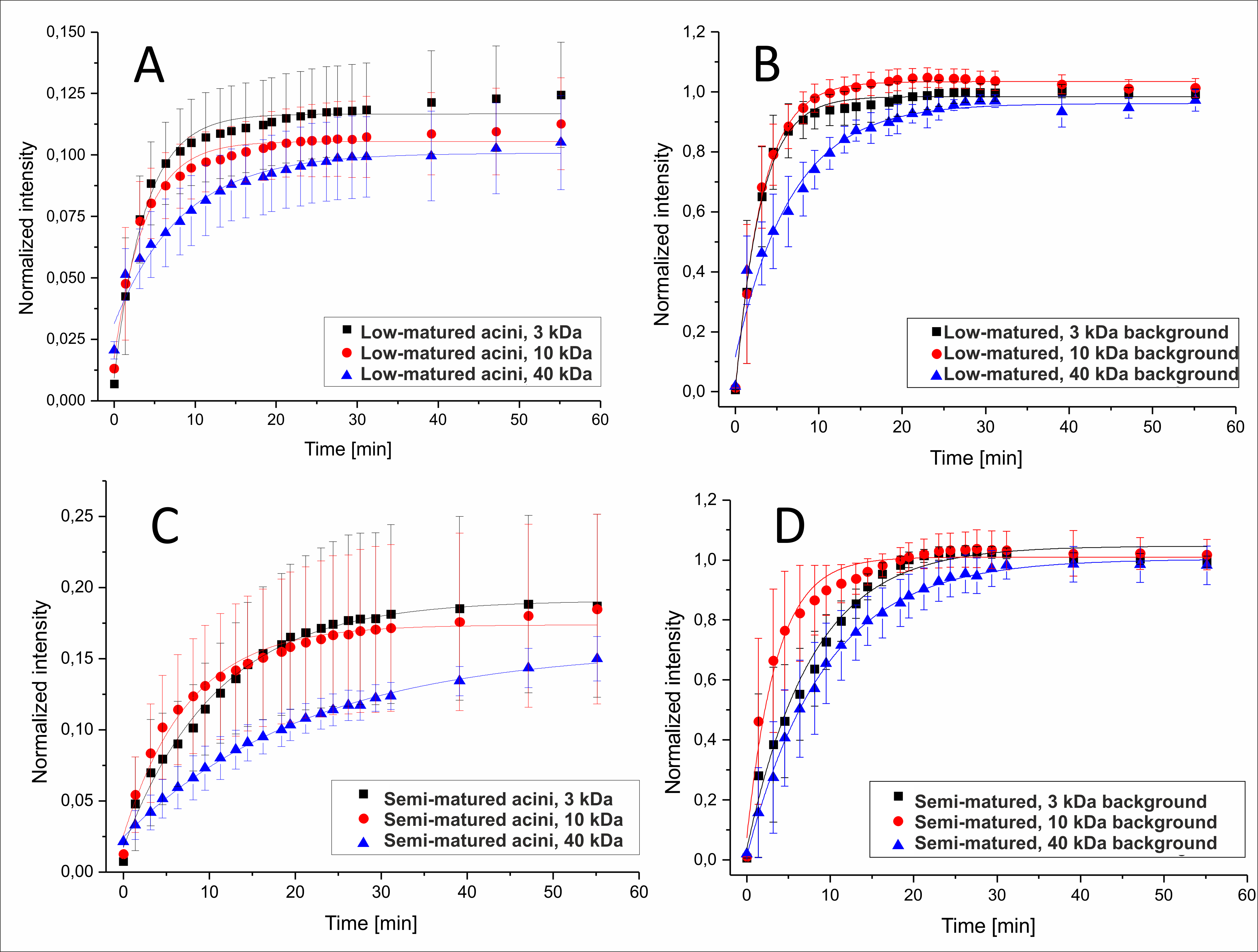

Supplement: S2 Fig — Average fluorescence intensity profiles recorded in (A.) low-matured acini and (B.) corresponding background ROIs and (C.) semi-matured acini and (D.) their background ROIs. Normalizations are with respect to the background plateau for each molecular weight. Bars indicate s.d. The superimposed curves are fits of S Eq. 1. For details of region identification see S1 Protocol. (TIF) [file pone.0145174.s002.tif]

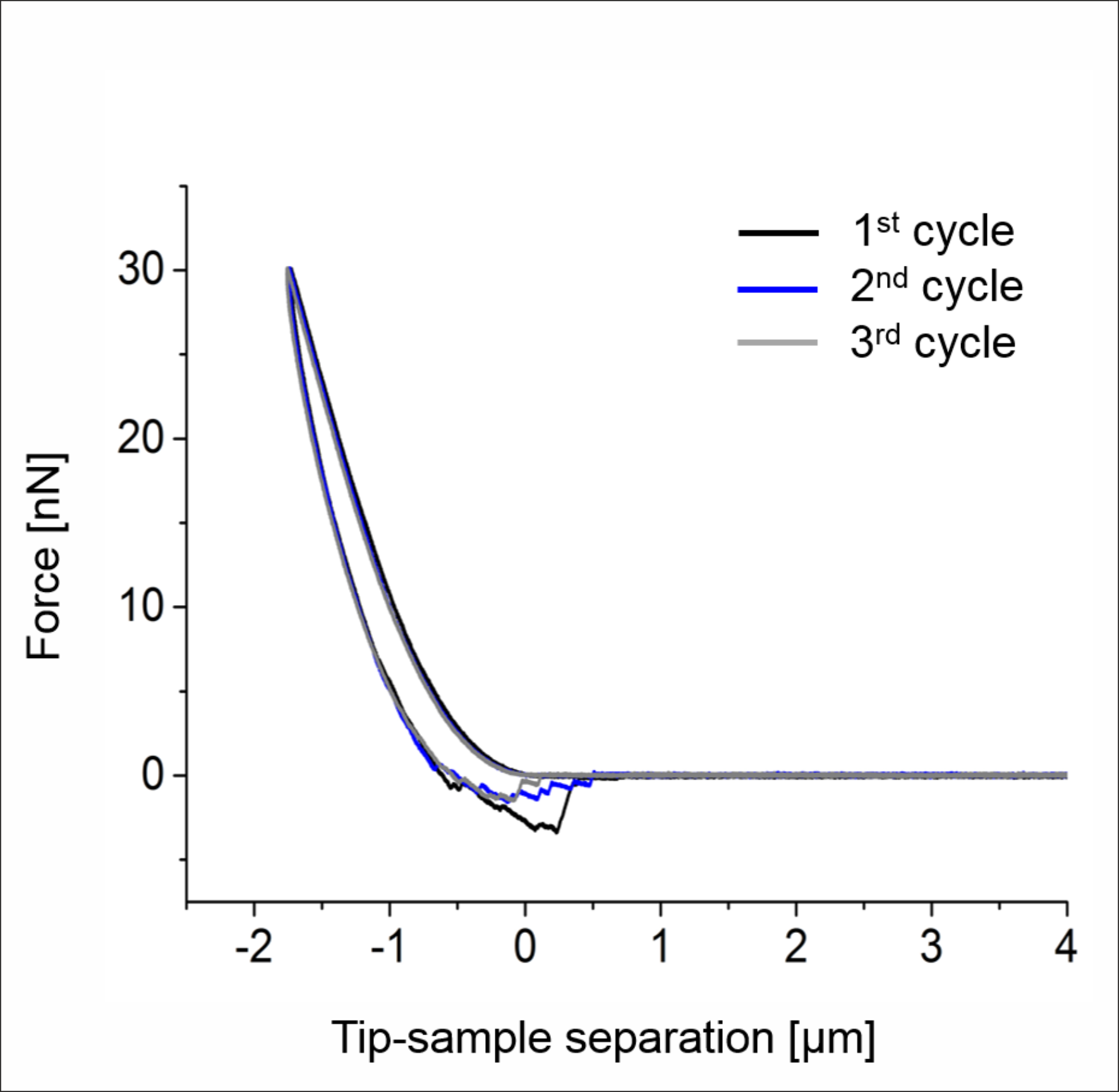

Supplement: S3 Fig — The plot shows three consecutive AFM indentation cycles that were performed on a native MCF-10A acinus. The almost perfect overlap of successive curves indicated the absence of plastic deformation and an overall elastic behavior of the acinar structures under this deformation. (TIF) [file pone.0145174.s003.tif]
