# Supplementary material for: The Acinar Cage: Basement Membranes Determine Molecule Exchange and Mechanical Stability of Human Breast Cell Acini
Source: PLoS One. 2015 Dec 16;10(12):e0145174. doi: 10.1371/journal.pone.0145174 (PMC4684506; doi:10.1371/journal.pone.0145174)
Supplement: S1 Protocol — (PDF) [file pone.0145174.s004.pdf]

Protocol S1. Information about the masking algorithm and permeation data analysis.

To follow the fluorescence intensity profiles of MCF10A spheres, acinar domains were isolated from the background via a masking algorithm implemented in Matlab. First, all fluorescent images were median filtered with a filter size of 35x35 pixels: this led to a smooth image displaying a background and a foreground (sphere) part. To extract the boundary of the acinus, the gradient of the filtered image was calculated using Matlab's imgradient-function (Prewitt method). Masking the gradient image with its mean gray value as a threshold, the boundary of the sphere was identified. Due to border artifacts, the outer 20 pixels of the whole mask were set to zero. Subsequently, holes within the mask were filled and morphological closing was performed. If more than one structure was found, the largest was chosen as current sphere mask. The surface so identified also included the brighter basal membrane surrounding the sphere. To remove this section, the mean gray value of the image covered by the mask was again used as a threshold: every pixel with gray value higher than the threshold was discarded. Morphological closing and holes filling allowed to obtaining the final sphere mask.

Mean intensities of low-matured MCF10A acini were analyzed based on this mask. Given the high autofluorescence displayed by the semi-matured acini, though, an additional step was performed prior to their analysis. The median of the grey value distribution of the image covered by the final mask was used as a threshold to isolate the very bright domains (pixels brighter than the median): these pixels were discarded as autofluorescence, and the remaining part was analyzed. To determine the background intensity, ROIs (Region Of Interest) were manually marked in the image and the median gray values of these ROIs were calculated for each time frame.

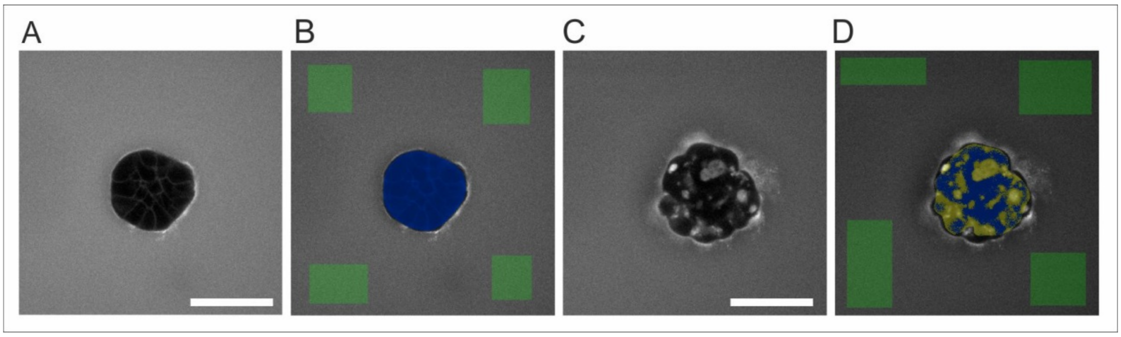

Protocol S1 Fig 1. Representative image processing using the masking algorithm.
A. Florescent image (TexasRed labelled dextran) of a low-matured MCF10A acinus isolated form the EHS gel substrate and **B.** mask used to measure the dextran tracer intensity profile within the acinus interior. Marked in green are the manually selected ROIs used for background calculation. **C.** Fluorescent image of an isolated semi-matured MCF10A acinus and **D.** corresponding mask. Yellow areas mark auto fluorescent domains that have been discarded from the analysis. Scale bars: 50 µm.

Permeation analysis

Assuming a simple model in which two compartments containing different concentrations of solute are divided by a semipermeable membrane, one can write down the equations for concentration equalization as follows:

$$C(t) = C_\infty + (C_0 - C_\infty)e^{-t/\tau} \quad \text{S Eq. 1}$$

Where, for a given compartment, $C(t)$ is the concentration over time, C_0 the initial one and C_∞ the final one. The time constant τ would then be linked to the permeability constant p of the membrane via $\frac{1}{\tau} = Ap\frac{V_1+V_2}{V_1V_2}$ (with A the membrane surface, and V_1 and V_2 the two compartment volumes). In principle, this would allow us to obtain p from fitting S Eq.1 to the intensity profile of the dextran tracers permeated within the MCF10A acini. In our case, though, the hypothesis of equilibration does not strictly hold, due to the constant cellular uptake of dextrans. Furthermore, neither the osmotically active volume V nor the surface area A can be determined to reasonable accuracy. Finally, also the change in concentration of the outer solution is not abrupt but occurs with a certain speed. Given the impossibility to extract p from our data, we fitted our experimental results with S Eq. 1 and used the time constants τ as semi-quantitative measure of permeability.
